# Motor cortex-based intracortical BCI performance is robust to changes in attentional load during dual-tasking

**DOI:** 10.1088/1741-2552/aebb65

**Published:** 2026-11-10

**Authors:** E Canario, C Shearer, M Akcakaya, D Weber, S Chase, J L Collinger

**Affiliations:** 1 Rehab Neural Engineering Labs, University of Pittsburgh, Pittsburgh, PA, United States of America; 2 Department of Bioengineering, University of Pittsburgh, Pittsburgh, PA, United States of America; 3 Department of Physical Medicine and Rehabilitation, University of Pittsburgh, Pittsburgh, PA, United States of America; 4 Department of Electrical & Computer Engineering, University of Pittsburgh, Pittsburgh, PA, United States of America; 5 Department of Mechanical Engineering, Carnegie Mellon University, Pittsburgh, PA, United States of America; 6 Neuroscience Institute, Carnegie Mellon University, Pittsburgh, PA, United States of America; 7 Department of Biomedical Engineering, Carnegie Mellon University, Pittsburgh, PA, United States of America

**Keywords:** brain-computer interface, intracortical, electroencephalography (EEG), motor cortex, attention, dual-task

## Abstract

*Objective.* High performance intracortical brain-computer interface (iBCI) control has been demonstrated in research settings, but performance can still vary within and between sessions. One potential source of this variability is the change in attentional load that comes from processing naturally occurring distractors such as thoughts, sounds, fatigue, or pain. To improve the consistency of iBCI performance in real-world environments where this sort of multi-tasking is inevitable, we must understand how shifts in attention can impact performance. *Approach.* Here we examined the effect of attentional load on iBCI performance and movement-related neural activity using a 2D cursor translation + click iBCI task paired with an *N*-Back working memory task to increase attentional load during dual-task performance. Two participants (P2 and P4) with tetraplegia completed the study while enrolled in a long-term clinical trial of an iBCI device. Common neural correlates of attention (theta and alpha band power) were measured with simultaneously recorded scalp electroencephalography (EEG). *Main Results.* While the EEG metrics and difficulty ratings suggested increased attentional load during dual-tasking, iBCI performance was quite robust across the various dual-tasking conditions. One participant, P2, experienced a small but significant increase in trial completion time and normalized path length during the mild attentional load condition compared to the moderate load condition. Signal quality differences between the two participants may have impacted the results, as P2 had lower signal quality and was therefore likely more vulnerable to attentional load. P4’s higher signal quality likely allowed him to accommodate increased attentional load without a drop in performance. *Significance.* Overall, iBCI performance appears to be robust to attentional load, but the complex trends observed here reflect a need for continued investigation of BCI use under different cognitive states to elucidate potential challenges and compensatory mechanisms across participants.

## 1. Introduction

Intracortical BCIs (iBCIs) have achieved a high level of performance in controlled laboratory environments (Collinger *et al* 2013b, Pandarinath *et al* 2017, Card *et al* 2024, 2026, Wilson *et al* 2026). This level of performance has motivated a transition to real-world testing in home environments in both academic and industry-led clinical trials (Card *et al* 2026, Neuralink 2026, Oxley 2025, Simeral *et al* 2021, Weiss *et al* 2019). However, assistive technologies are often abandoned by users due to reasons such as mismatched user needs and device function, difficulty of use, and poor performance (Phillips and Zhao 1993). Potential BCI users desire systems that are highly reliable and require minimal setup time (Huggins *et al* 2011, Collinger *et al* 2013a, Blabe *et al* 2015). Reliable and stable BCI control can be challenging to achieve because neural recordings are inherently unstable due to physical factors such as micro-motion of arrays (Perge *et al* 2013) or context-dependent changes in the underlying neural population activity (Perge *et al* 2013, Sussillo *et al* 2016, Tadipatri *et al* 2017). Some progress has been made using decoding algorithms that rely on neural networks to enable stable control with little to no recalibration (Card *et al* 2024, Jude *et al* 2025) but their robustness to changes in mental state or environment has not been fully evaluated.

In other motor control contexts, attention is known to impact task performance (Pashler 1994, Engström *et al* 2017, Draheim *et al* 2022), but this has been less investigated in BCI. Furthermore, the few studies of attention focus primarily on BCIs that rely on scalp electroencephalography (EEG), with few examining the effect of changes in attention on implanted BCI control. In contrast to iBCIs that record activity from small groups of neurons near each electrode tip, EEG BCIs record aggregate neural activity from large populations of neurons with electrodes placed on the scalp. BCIs based on motor imagery often use sensorimotor rhythm activity to drive the movement of an end effector, such as a computer cursor. In one study of EEG BCI-controlled cursor movements, performance was found to be sensitive to speech-related distractors, which decreased target acquisition and path efficiency (Foldes and Taylor 2013). Another EEG-BCI study found that distractors of various cognitive loads impacted lower-performing participants but not higher-performing ones (Emami and Chau 2020). The impact of attentional load has been less studied for iBCIs, but some evidence suggests that iBCIs may be more robust to distractors than EEG-based BCIs, with only minimal performance loss during simple motor tasks performed with various distractions (Guthrie *et al* 2021). However, a limitation of this previous study is that the BCI task was relatively simple, as participants only had to move between two nearby targets repeatedly, so it is unclear how attention may impact iBCI use particularly for more complex and realistic tasks. Evidence from attentional studies in other contexts such as driving finds that tasks that are more difficult, requiring active thought and increased effort, are more affected by distractors (Engström *et al* 2017). One iBCI study found that cursor control completion times increased during continuous speech but not shorter forms of speech (Stavisky *et al* 2020). This suggests that more involved distractor tasks have a greater effect on BCI control.

In addition to impacting motor control performance, cognitive factors can also directly impact the neural activity that drives movement. For example, distractors can disrupt the sensorimotor rhythm desynchronization often used to drive EEG-BCIs, even if there is no effect on performance (Emami and Chau 2018). Arousal can modulate brain-wide neural activity (Musall *et al* 2019, Stringer *et al* 2019, McCormick *et al* 2020) including motor activity during BCI task learning (Hennig *et al* 2021). Further, motor cortical activity can change depending on expected reward or motivational state (Ramkumar *et al* 2016, Ramakrishnan *et al* 2017, An *et al* 2019, Smoulder *et al* 2024).

To better understand how motor-related neural signals and BCI performance change with attentional load, we recorded scalp EEG during iBCI use. Neural correlates of attention are commonly measured with EEG during concurrent motor tasks. For example, EEG recordings showed increased frontal area theta band power and decreased parietal area alpha band power while participants were driving under distracting conditions (Wang *et al* 2018). Similar trends were observed during dual task performance involving a motor and working memory (*N*-Back) experiment (Ozdemir *et al* 2016) and during a dual task standing and verbal math experiment (Kahya *et al* 2022). Frontal theta power (∼4–7 Hz) is thought to be involved in executive function during cognitive tasks (Cavanagh and Frank 2014). Alpha power (∼8–12 Hz) in the parietal-occipital region is thought to be involved in the allocation of cortical resources to internal or external tasks (Magosso *et al* 2019).

Here we aimed to investigate the impact of attentional load on iBCI performance and the underlying movement-related neural activity in two human iBCI users while utilizing EEG to measure metrics of attention across the brain. Participants used the iBCI to perform a complex cursor control task involving click and drag actions (Dekleva *et al* 2021) combined with the *N*-Back working memory task (Kirchner 1958) to increase attentional load, emulating real-world distractions. The *N*-Back task impacted attention but had little effect on BCI performance. One participant had been implanted for a much longer duration and therefore had lower signal recording quality and baseline BCI performance. Despite these differences, iBCI cursor control was generally robust to attentional load. Changes in movement-related neural activity were very small in magnitude across conditions, suggesting that little compensation was required. We demonstrated the feasibility of concurrent EEG recording and iBCI control, which may enable future studies that wish to examine how iBCIs generalize to different settings, where the user may experience stressors, such as pain or fatigue, that impact their ability to attend to BCI use.

## 2. Materials and methods

### 2.1. Data collection

Two participants, P2 (28 years old at implant, C5 ASIA B spinal cord injury) and P4 (31 years old at implant, C4 ASIA A spinal cord injury), with tetraplegia who were participating in a clinical trial of an iBCI device for restoring upper limb function completed the experiments for this project. This study was conducted under an FDA Investigational Device Exemption (NCT01894802) in accordance with the principles embodied in the Declaration of Helsinki and approved by the University of Pittsburgh Institutional Review Board. Informed consent was obtained prior to any experimental procedures. Study participants each had two intracortical microelectrode arrays (NeuroPort Electrodes, Blackrock Neurotech, Salt Lake City, UT) implanted in the motor cortex (88 channels per array for P2 and 96 for P4) and two 32 channel arrays in the somatosensory cortex. P2 was implanted approximately 8 years prior to data collection, while P4 was implanted approximately 6 months prior to data collection.

Intracortical signals were collected at 30 kHz using the NeuroPort System (Blackrock Neurotech) and filtered with a 1st order 750 Hz high-pass filter. Threshold crossing events (−4.5 RMS) were recorded and binned (every 20 ms) to estimate spike rates. The binned spike counts were then smoothed with an exponential smoothing function with a 440 ms window. To isolate local field potentials (LFPs), the 30 kHz data were low-pass filtered with a 250 Hz 4th order filter and then down-sampled to 1 kHz. We calculated the mean channel raw beta band frequency power (13–30 Hz) from each trial. This was done by first calculating a short-time Fourier transform for each trial and then averaging across time within a specific beta band. A few trials (3 for P2, 2 for P4) were rejected due to software error resulting in no intracortical data. Figure 1(A) displays the approximate location of arrays (represented by blue squares) and percutaneous pedestal connectors (represented by the blue circles).

**Figure 1.**
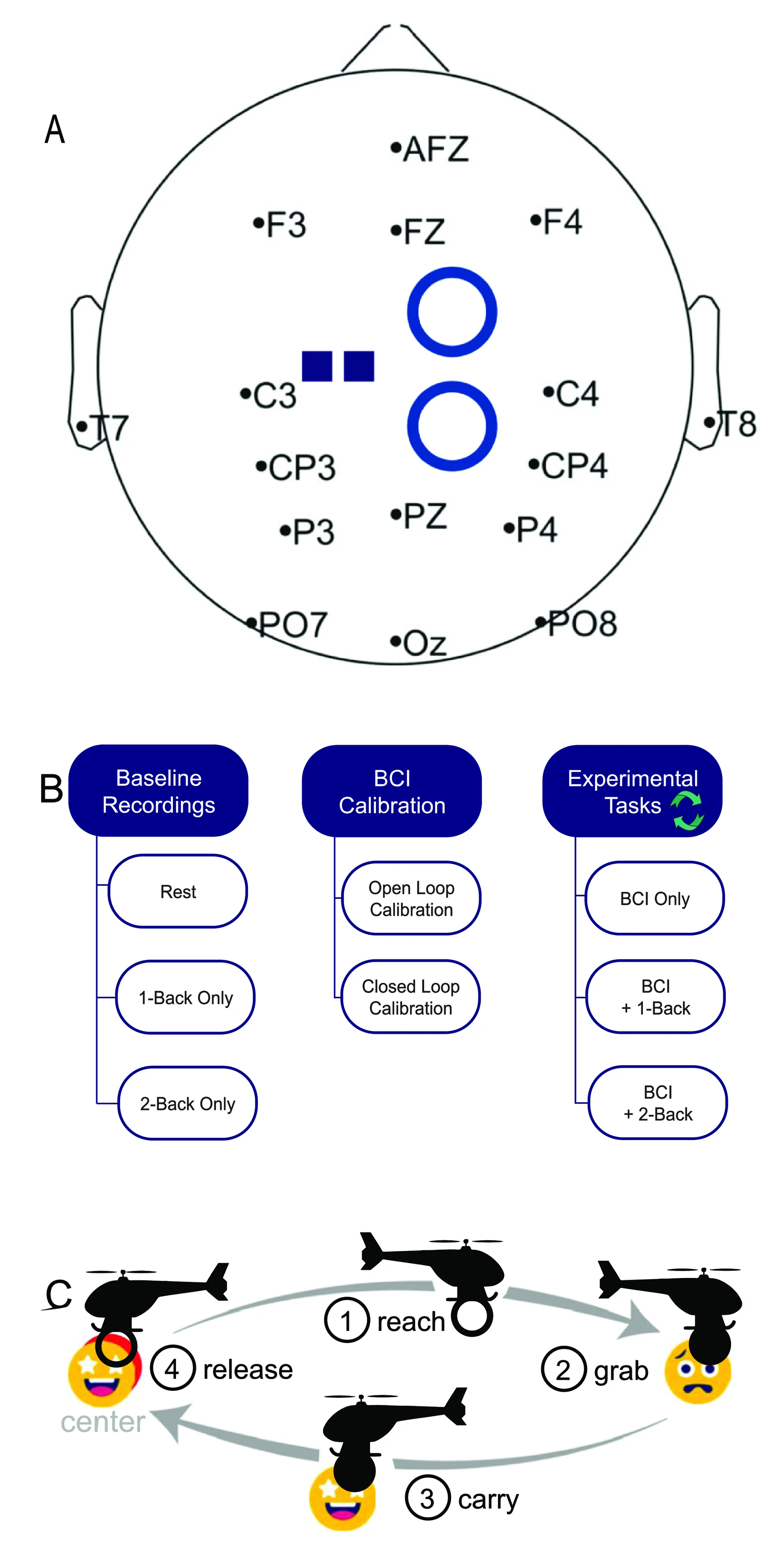
(A): Location and names of the 16 EEG channels. Blue ovals indicate the approximate location of percutaneous pedestal connectors and blue squares indicate the approximate location of intracortical motor arrays on the brain. Frontal electrodes are marked by the letters F and AFZ. Parietal-occipital electrodes are labeled with the letter P, PO, or O. Central electrodes are labeled with C or CP, and the T label indicates electrodes on the temporal region. (B): Each session consisted of baseline recordings, BCI calibration, and then BCI control with and without N-Back. Baseline recordings included three minutes of rest, 1-Back only, and 2-Back only. The dual-tasking experimental conditions were block randomized each session. (C): BCI task phases. First, during the reach phase the participant navigated the cursor (helicopter) to a target (face emoji). Next, they clicked to grab the target and carry it back to center where they ‘unclicked’ to release the target. Trials were separated by a 2 s intertrial interval.

To provide a more global measurement of neural activity, EEG data were collected using 16 g.tec ladybird active electrodes and a g.USBamp biosignal amplifier (g.tec medical engineering, Schiedlberg, Austria) at 256 Hz. The right mastoid was used as the reference electrode, and the left mastoid was used as ground. Channel locations are shown in figure 1(A) derived from the international 10–20 system of EEG electrode placement (Jasper 1961) with slight adjustments to accommodate the percutaneous pedestals that allow connection to the implanted intracortical electrodes. EEG data were recorded in BCI2000 (Schalk *et al* 2004) and processed in EEGLAB (Delorme and Makeig 2004). EEG data were filtered with a 0.5-55 Hz bandpass and then bad channels were visually identified and removed. At most, 1–2 channels were removed per set. Next, we used artifact subspace reconstruction (Mullen *et al* 2015) with a cutoff of 15 standard deviations to automatically identify bad data segments and remove them. We then performed a common average reference with all remaining channels. Finally, ICA was performed to remove eye-related components that might contaminate the data. To avoid discontinuities, individual trials were rejected if they contained any rejected data segments. For data collected while the participant was at rest or performing *N*-Back alone, this pipeline resulted in 19%–25% (P2) or 21%–41% (P4) of the data being rejected. For BCI tasks and dual-tasking, 1%–5% (P2) or 35%–48% (P4) of trials were rejected. More trials were rejected for P4 due to greater noise for reasons such as more movement artifacts and worse electrode contact with the scalp. Table 1 indicates the total number of EEG trials per condition per participant after rejection.

**Table 1.** Number of EEG trials included for analysis for each condition. Excluded data were rejected due to artifact contamination.

Participant	Rest	1-Back	2-Back	BCI Only	BCI + 1-Back	BCI + 2-Back
P2	295/363 (81%)	258/342 (75%)	265/336 (79%)	143/144 (99%)	144/147 (97%)	153/160 (95%)
P4	264/386 (69%)	305/385 (79%)	224/378 (59%)	152/232 (65%)	118/208 (56%)	109/208 (52%)

### 2.2. Session design

Figure 1(B) shows the overall session design that included baseline recordings, BCI calibration, and experimental dual-tasking conditions involving BCI and the *N*-Back working memory test (Kirchner 1958). Participants were familiarized with the *N*-Back through practice trials before any experimental conditions were recorded. The *N*-Back is a task designed to test working memory. In the *N*-Back conditions, participants were presented with letters via audio with an inter-trial interval of 2.5 s while they performed the BCI task. Participants were instructed to verbally indicate when the letter presented matched the letter presented *N* (where *N* = 1 or 2) letters ago. The presentation of letters was randomized. An example of 2-Back would be: I G K **G** B L, where the subject would say ‘match’ on the bolded letter, G. As *N* increases, subjects are required to hold more information in their working memory, thus presenting a greater challenge to attentional load. As with many dual-task studies, we expected that managing this secondary task required more attentional resources from participants due to the greater attentional load compared to only doing one task (Pashler 1994, Finley *et al* 2014).

At the beginning of each session, we recorded EEG and intracortical data during a three-minute resting baseline period. Participants were instructed to look at a fixation cross while minimizing movement. We also collected EEG and intracortical data during performance of the 1-Back and 2-Back conditions without any BCI control, each for 3 min. We then calibrated the BCI decoder and proceeded to the experimental conditions (See *Task Design* for details). EEG and intracortical neural data were recorded while the participants performed one of three conditions: 1) BCI Only, 2) BCI with mild attentional load (BCI + 1-Back), and 3) BCI with moderate attentional load (BCI + 2-Back). Participants were told to focus on the BCI cursor task as the primary task but to try their best to complete the *N*-Back accurately. The condition order was block randomized each session to minimize order effects. We collected 1–2 sets of 16–24 trials of each condition depending on time constraints (table 1). After each condition, participants were asked to rate their mental effort on a scale from 1–10 (Paas 1992). We collected 5 sessions with P2 and 6 with P4.

### 2.3. Task design

To calibrate the BCI decoder, we began with open-loop calibration where the participants attempted to perform a center-out-and-back cursor control task in both clicked and unclicked states (Dekleva *et al* 2021). Neural data was recorded while the cursor movement was controlled by the computer. Factor analysis, a type of dimensionality reduction, was used to produce a 20-dimensional neural state space. This state space was used to train a Kalman filter to decode 2D cursor velocity and two LDA classifiers to decode click and unclick. We repeated the process with a partially assisted closed-loop calibration in which off-target movements were attenuated to train a second decoder that was then used for the remainder of the session.

Subjects performed an iBCI-controlled cursor task, which was a gamified center-out grasp and carry task (figure 1(C)). This 2D +click BCI task was designed to emulate mouse use on a computer. It consisted of an initial presentation phase where the target was presented, a reach phase where participants reached towards the target, a click phase where they clicked the target, and a center phase where they returned to the center to end the trial. A black screen was then shown for two seconds to serve as an intertrial rest period. Participants had a maximum of 20 s to complete each phase (i.e. reach, click, and return to center) where failure in any phase meant failing the trial. For the reach phase the participant needed to move the cursor to the target, such that the middle of the cursor (radius= 0.02 a.u.) overlapped with the target (radius= 0.04 a.u.). While touching the target, the participant had to successfully click the cursor and then maintain the click in order to bring it back to the center (overlapping with the target). Early drops were considered failures, but early clicks were not. There was no hold time in any phase. A typical block consisted of 8 trials, with 2 or 3 blocks per set. The order of the sets was randomized for each session.

BCI task performance measures included success rate (expressed as the percentage of trials correct for each block of 8 trials) and reach phase completion time, total trial completion time, and normalized path length for the successful trials. Normalized path length was calculated as the ratio of the actual cursor path length to the ideal cursor path length (where a straight line to the target is equal to 1) during the reach phase. Total trial completion time was included to assess impact on the full trial with translation and clicking, while reach phase completion time focuses on the simpler translation period.

### 2.4. Neural data analysis

We extracted the attention-related measures of EEG band power for the theta (4–7 Hz) and alpha (8–12 Hz) bands as well as the sensorimotor rhythm (beta band, 13–30 Hz). We first calculated the event-related spectral perturbation (ERSP) for every trial using EEGLAB, and computed the average power in the theta, alpha, and beta bands for the first two seconds of the reach phase, obtaining a trial level measure that captured the initial period of movement towards a target before participants began self-correcting in response to error. We focused on the first two seconds of movement because the intended kinematics are expected to be consistently pointed towards the target during this time. After this time, the subjects may begin to make corrective movements thus making it difficult to distinguish changes in movement intention from changes related to attentional load. EEG features of attention are present broadly in the brain but are strongest in certain regions, such as alpha primarily appearing in the parietal region and theta in the frontal (Emami and Chau 2020). We used a region of interest approach and averaged power in the parietal-occipital channels (PO7, PO8, P4, P3, Pz) to obtain alpha power and frontal area channels (AFz, Fz, F3, and F4) to obtain theta power as is common in studies of attention (Scharinger *et al* 2017). Beta band power is expected to decrease during movement periods so a smaller value of beta band power during the reach phase would indicate stronger movement-related activity. For examples of the EEG signal, see figure S1 in the supplementary material. From the intracortical spike rate data, we calculated the mean firing rate in each trial (consisting of the first two seconds after reach begins, as in the EEG processing) for all conditions (Wilson *et al* 2026). We also computed the neural reaction time based on the time it took to reach the peak firing rate during the first two seconds of the trial’s reach phase. We also computed beta band power from the intracortical LFP recordings. We first calculated the ERSP for every trial across the first two seconds of reach. We averaged this across all motor channels (176 for P2, 192 for P4). Finally, beta band power was then averaged across frequencies and time to obtain a trial level metric of the LFP beta band power.

### 2.5. Statistical testing

Data were combined across days for statistical testing. Normalized path length, total trial and reach phase completion times were found to have skewed distributions so they were log-transformed to aid normality. For every metric of performance, attention, and motor signal, we ran separate statistical tests using these measures as dependent variables. First, we fit a linear mixed effects model using R to evaluate the fixed effect of condition and random effect of session. From this model, we report the standard deviation of the random effect of session. If session had a significant impact on a given outcome measure, we computed the intraclass correlation coefficient (ICC) to quantify how much variance was attributed to this random effect. We then assessed the significance of the linear mixed effects model with an ANOVA based on the variance explained by condition after accounting for the random effects. If the main effect of condition was significant based on the ANOVA, we performed post-hoc comparisons between conditions using the Tukey method on the estimated marginal means from the linear mixed effects model. For significant post-hoc comparisons, we report the difference in estimated marginal means between conditions, alongside the standard error, and the effect size (calculated as Cohen’s d from the estimated marginal means and the population standard deviation). We also report the random effect of session when condition was significant, reporting the standard deviation and the ICC as a proxy for the effect of session on outcomes.

## 3. Results

### 3.1. iBCI performance is stable under increasing attentional load

iBCI performance was generally robust to attentional load challenges induced by the dual-tasking *N*-Back paradigm (figure 2). Neither participant showed significant differences in success rate (P2: F(2, 49.033) = 0.848, *p* = 0.435; P4: F(2, 73.250) = 1.676, *p* = 0.194) or reach phase completion time (P2: F(2, 385.390) = 2.701, *p* = 0.068; P4: F(2, 629.330) = 1.307, *p* = 0.271). P2 had higher total trial completion time in BCI + 1-Back compared to BCI + 2-Back (log transformed mean difference = 0.080, SE = 0.024, *p* < 0.05, effect size = 0.421), but P4 exhibited no differences (P2: F(2, 385.040) = 5.751, *p* = 0.003; P4: F(2, 629.180) = 0.405, *p* = 0.667). For P2’s total trial completion time, the random effect of session accounted for a moderate portion of the variance (SD = 0.172, ICC = 0.446). Additionally, P2 also had an increase in trial normalized path length in BCI + 1-Back as compared to BCI + 2-Back (log transformed mean difference = 0.058, SE = 0.019, *p* < 0.05, effect size = 0.381) but this was not the case in P4 who had no significant differences (P2: F(2, 385.280) = 4.739, *p* = 0.009; P4: F(2, 629.760) = 0.793, *p* = 0.453). For P2’s trial normalized path length, the random effect of session accounted for a small portion of the variance (SD = 0.069, ICC = 0.160). Dual-tasking was rated as more difficult than BCI control alone (figure S2, supplementary material). *N*-Back accuracy rates remained high during dual-tasking (P2: BCI + 1-Back Acc = 0.991, BCI + 2-Back Acc = 0.976; P4: BCI + 1-Back Acc = 0.899, BCI + 2-Back Acc = 0.895) indicating that participants did not simply ignore the secondary task. For reference, performance during single-task performance of the *N*-Back was similar (P2: 1-Back Acc = 1.00, 2-Back Acc = 0.981; P4: 1-Back Acc = 0.923, 2-Back Acc = 0.856).

**Figure 2.**
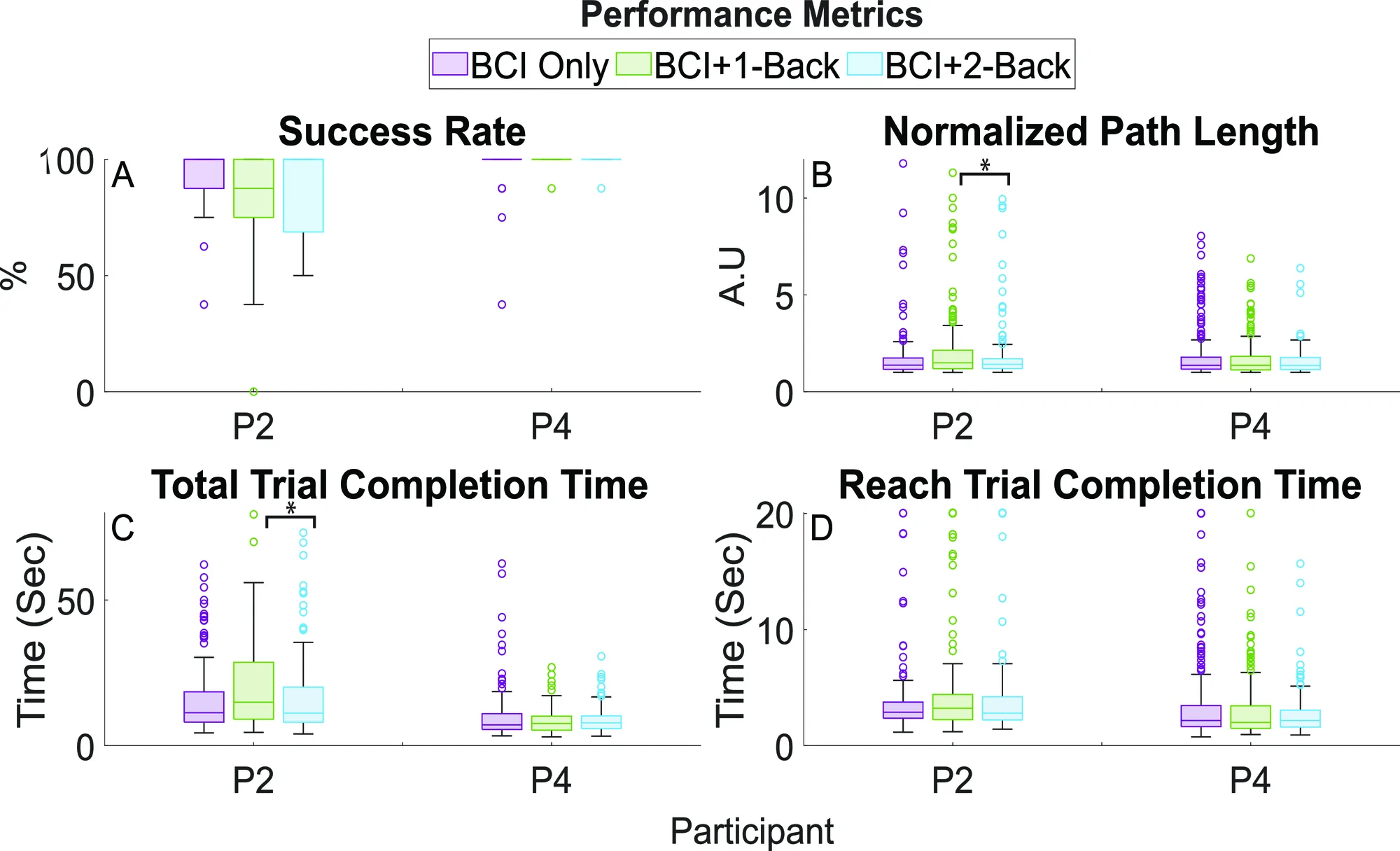
Performance metrics during dual-tasking conditions. (A): Success Rate, (B): Normalized Path Length, (C): Total Trial Completion Time, (D): Reach Trial Completion Time. P2 exhibited a higher path length and total trial completion time in BCI + 1-Back compared to BCI + 2-Back (p <0.05, Tukey post-hoc pairwise comparisons), indicating worse performance.

### 3.2. Neural correlates of attention are modulated by attentional load

EEG metrics of attention were well modulated by performance of the *N*-Back task (without BCI control) as compared to rest. Theta power was significantly different among the three baseline conditions (rest, 1-back, & 2-back) for P2 and P4 (figure 3; P2: F(2, 812.670) = 168.080, *p* < 0.001; P4: F(2, 807.400) = 22.784, *p* < 0.001). For P2, theta power was higher in 1-Back compared to rest (mean difference = 1.919, SE = 0.105, *p*< 0.05, effect size = 1.574) or 2-Back (mean difference = 0.894, SE = 0.107, *p* < 0.05, effect size = 0.733), while 2-Back was also higher than rest (mean difference = 1.024, SE = 0.103, *p* < 0.05, effect size = 0.840). In P4, theta power was higher in 1-Back compared to rest (mean difference = 1.095, SE = 0.167, *p* < 0.05, effect size = 0.562) or 2-Back (mean difference = 0.745, SE = 0.169, *p* < 0.05, effect size = 0.382). The random effect of session accounted for a small to moderate portion of the variance in each participant (P2: SD = 0.616, ICC = 0.167; P4: SD = 1.361, ICC = 0.325).

**Figure 3.**
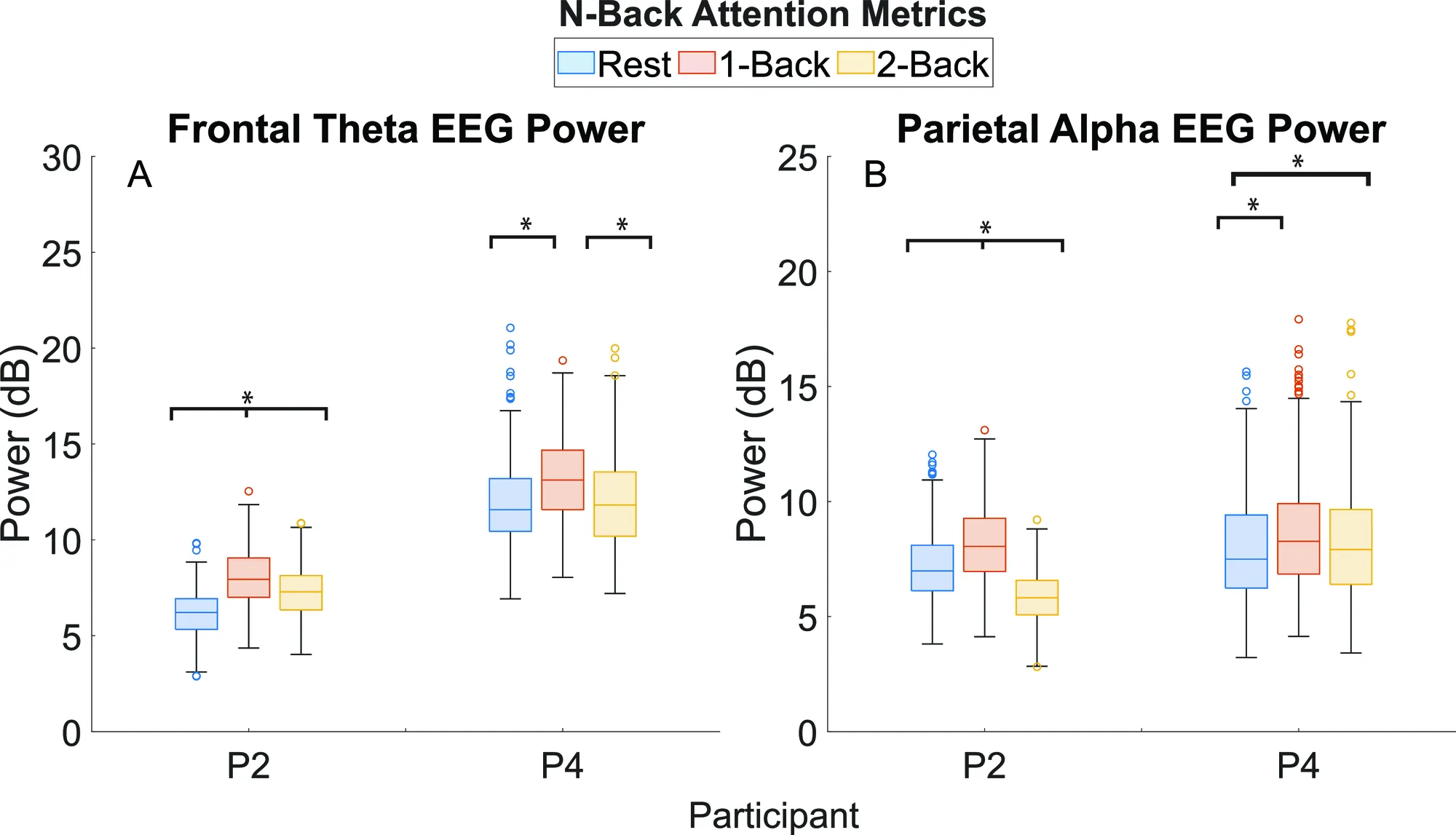
Comparing N-Back only to resting state. (A) Frontal Theta EEG Power, (B) Parietal Alpha EEG Power. Participants displayed higher theta power in 1-Back compared to rest (p <0.05, Tukey post-hoc pairwise comparisons), with P2 also having higher theta power in 2-Back (p <0.05, Tukey post-hoc pairwise comparisons). Participants displayed higher alpha power in 1-Back compared to rest (p <0.05, Tukey post-hoc pairwise comparisons), with P2’s 2-Back alpha power being lower than other conditions (p <0.05, Tukey post-hoc pairwise comparisons) but P4’s 2-Back also having higher alpha power than Rest (p <0.05, Tukey post-hoc pairwise comparisons).

Alpha power was also higher during the 1-Back condition as compared to rest (mean difference = 1.030, SE = 0.123, *p* < 0.05, effect size = 0.725) and 2-Back (mean difference = 2.270, SE = 0.125, *p* < 0.05, effect size = 1.595) in P2, with rest having higher alpha power than 2-Back (mean difference = 1.240, SE = 0.121, *p* < 0.05, effect size = 0.870). In P4 it was lower in rest compared to 1-Back (mean difference = 1.070, SE = 0.165, *p* < 0.05, effect size = 0.555) and 2-Back (mean difference = 0.913, SE = 0.173, *p*< 0.05, effect size = 0.473) (P2: F(2, 813.140) = 165.110, *p* < 0.001; P4: F(2, 807.200) = 23.539, *p* < 0.001). This result was in the opposite direction expected, as we initially hypothesized there would be a linear decrease in response to increasing attentional load. Thus, in effect, both alpha and theta (for both participants) seemed to increase the most in 1-Back but then decrease in 2-Back compared to 1-Back and/or rest. The random effect of session accounted for a small to moderate portion of the variance in each participant (P2: SD = 0.515, ICC = 0.089; P4: SD = 1.951, ICC = 0.475).

In dual-tasking (BCI + *N*-Back), we saw similar trends. Specifically, when looking at neural correlates of attention (figure 4), we saw changes in frontal theta power for both participants across dual-tasking conditions (P2: F(2, 433.110) = 6.243, *p =* 0.002; P4: F(2, 371.500) = 4.609, *p =* 0.011). For P2, theta power was higher in the BCI + 1-Back compared to BCI Only (mean difference = 0.440, SE = 0.133, *p* < 0.05, effect size = 0.390) and BCI + 2-Back (mean difference = 0.362, SE = 0.131, *p* < 0.05, effect size = 0.320). For P4, theta power was higher in the BCI + 2-Back condition as compared to BCI Only (mean difference = 0.887, SE = 0.318, *p* < 0.05, effect size = 0.373). The random effect of session accounted for a small to moderate portion of the variance in each participant (P2: SD = 0.742, ICC = 0.298; P4: SD = 1.766, ICC = 0.360).

**Figure 4.**
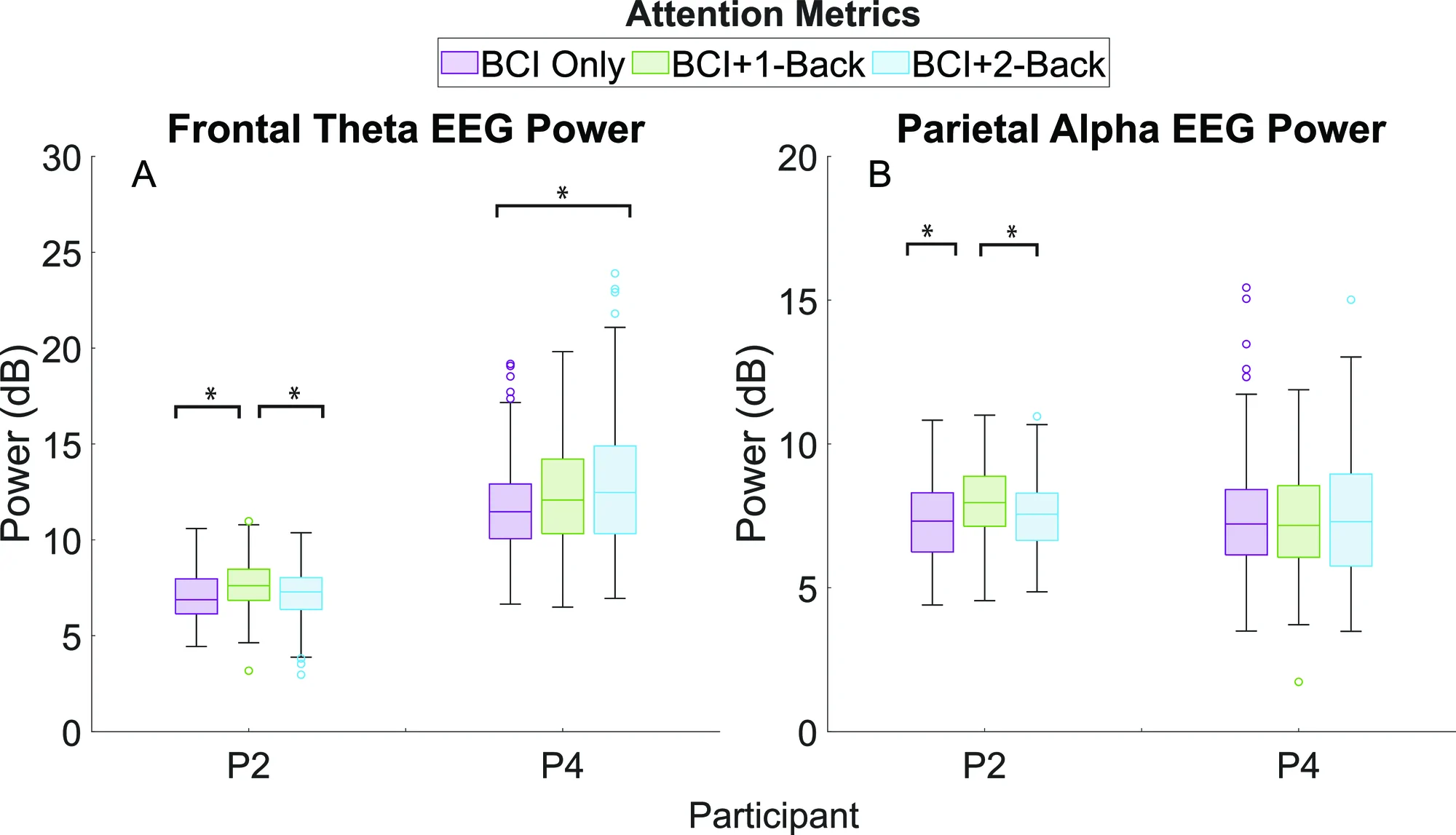
Comparing dual-tasking to BCI Only. (A): Frontal Theta EEG Power, (B) Parietal Alpha EEG Power. Participants displayed higher theta power in either BCI + 1-Back (P2) or BCI + 2-Back (P4) compared to BCI Only (p <0.05, Tukey post-hoc pairwise comparisons). P2 displayed higher alpha power in BCI + 1-Back compared to BCI Only and BCI + 2-Back (p <0.05, Tukey post-hoc pairwise comparisons).

When looking at alpha power in the parietal region, we found significant difference across conditions for P2 but not P4 (P2: F(2, 433.140) = 10.271, *p* < 0.001; P4: F(2, 372.210) = 0.6744, *p =* 0.523). For P2, alpha power was higher in the BCI + 1-Back condition compared to BCI Only (mean difference = 0.612, SE = 0.135, *p* < 0.05, effect size = 0.534) and BCI + 2-Back (mean difference = 0.337, SE = 0.133, *p* < 0.05, effect size = 0.294). The random effect of session accounted for a small portion of the variance for P2 (SD = 0.609, ICC = 0.212).

### 3.3. Minimal changes in movement-related signals under attentional load

We analyzed three movement-related signals for both participants to examine whether or not attentional load impacts the high-level intention signal in motor cortex. We found that movement-related activity was generally consistent across conditions (figure 5), mirroring the trends in BCI performance (figure 2). Neither participant showed differences in the EEG beta band power (P2: F(2, 433.160) = 1.660, *p* = 0.191; P4: F(2, 371.710) = 2.066, *p* = 0.128). Only P2 demonstrated any difference in the intracortical LFP beta band power (P2: F(2, 441.210) = 3.634, *p* = 0.027; P4: F(2, 638.370) = 0.982, *p =* 0.375), with BCI + 2-Back LFP beta band power being significantly lower than BCI Only (mean difference = 0.248, SE = 0.116, *p* < 0.05, effect size = 0.290). We examined raw beta band power during the reach phase (vs normalized to rest) so an increase in power signifies less beta band desynchronization and therefore less movement-related modulation. The random effect of session accounted for a small portion of the variance for P2 (SD = 0.455, ICC = 0.212). Only P4 showed a significant difference in the intracortical firing rate (P2: F(2, 441) = 2.530, *p* = 0.081; P4: F(2, 638.110) = 4.542, *p* = 0.011), where firing rate was higher during BCI + 2-Back compared to BCI Only (mean difference = 0.652, SE = 0.231, *p* < 0.05, effect size = 0.271) or BCI + 1-Back (mean difference = 0.555, SE = 0.236, *p* < 0.05, effect size = 0.231) signifying a stronger movement-related signal. The random effect of session accounted for a moderate portion of the variance for P4 (SD = 2.199, ICC = 0.448). Finally, neither participant exhibited differences in neural reaction time across conditions (P2: F(2, 443.270) = 2.480, *p* = 0.084; P4: F(2, 638.710) = 0.856, *p =* 0.425). While statistically significant, the magnitude of change in LFP beta band power for P2 and firing rate for P4 was very small suggesting that minimal compensation was required.

**Figure 5.**
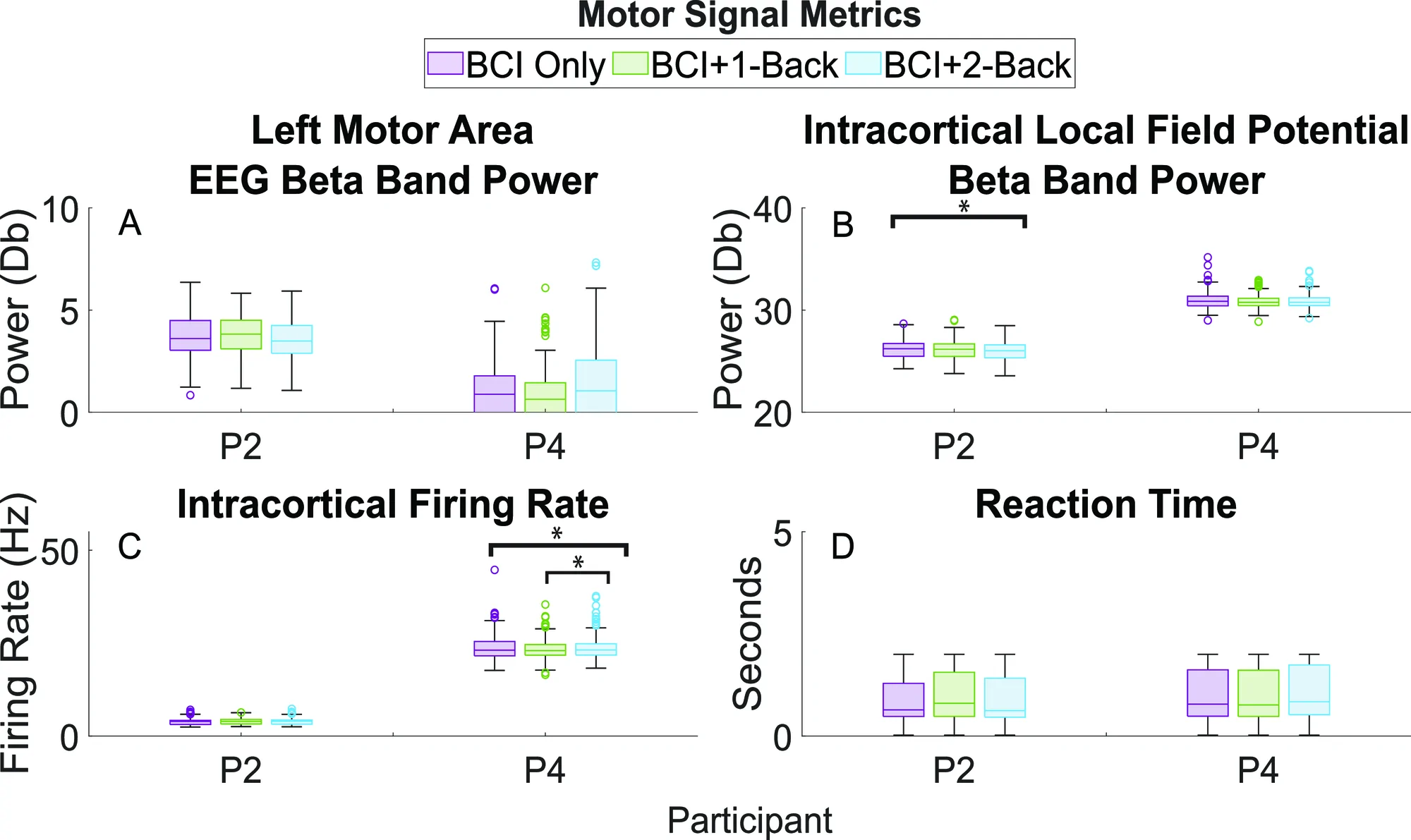
Measures of motor related activity from EEG and intracortical recordings, including (A): EEG beta band power, (B): Intracortical LFP beta band power, (C) Intracortical Firing Rate, (D) Neural Reaction Time. P2 exhibited a small but statistically significant decrease in intracortical LFP beta band power in BCI + 2-Back compared to BCI only, indicating an increase in the motor signal. P4 exhibited a small but statistically significant increase in intracortical firing rate in BCI + 2-Back compared to BCI Only and BCI + 1-Back (p <0.05, Tukey post-hoc pairwise comparisons), signifying an increase in the motor signal.

## 4. Discussion

This study explored the effects of attentional load on iBCI performance and attention- and movement-related neural signals. The *N*-Back task had little impact on iBCI performance even though it did cause changes in EEG correlates of attention. iBCI cursor control performance remained robust to attentional load even in a realistic and complex task involving click and drag actions. Movement-related neural activity measured intracortically and with EEG also remained stable during dual-tasking conditions when compared to BCI alone.

### 4.1. Performance is robust to attentional load

Participants exhibited few changes in performance across the different conditions, with only P2 displaying a slight increase in normalized path length in BCI + 1-Back and a more moderate increase in total trial completion time as compared to BCI + 2-Back. While such robustness is not unprecedented in the literature, the high level of attentional load here makes this nonetheless surprising. We built on previous iBCI work related to the impact of distractions (Guthrie *et al* 2021) by utilizing a more complex task design (that included target-guided reaching and grasping to control a virtual computer mouse vs only simple left/right translational movement), expecting a greater impact of attentional load on performance. Our results support the idea that iBCI control is robust across multiple levels of attentional load.

The observed performance robustness is striking when comparing iBCI to EEG-BCI. Studies of EEG BCIs have found comparatively greater effects of attention. In one study, there was a large effect (${\eta_p^2}$ = .31) that led to a 20% drop in BCI success rate during a triple task of 1D BCI control, maintaining cruise altitude on a flight simulator, and performing attentional tasks (Vecchiato *et al* 2016). In another study, EEG-BCI classification accuracy dropped by 5%–10%, in a BCI +speech task, depending on the classifier used (İşcan and Nikulin 2018). Emami and Chau (2020) showed a range of performance of nearly 30% within low-performance BCI users as cognitive load increased. (Foldes and Taylor 2013), also show a decrease of up to 20% in BCI success rate during high cognitive load, along with slightly worse trial completion times and path efficiency. By contrast, our worst performing participant, P2, had a success rate drop of less than 10% on average.

The reason for these differences likely arises from the signal source, with EEG being a coarse grained, non-spatially specific signal in comparison to highly specific movement-related information that can be measured with intracortical electrodes in motor cortex. Additionally, EEG BCI users often report that a significant amount of mental effort is required to use the BCI so dual-tasking may have more of an impact when the attentional load of single tasking is higher (Cao *et al* 2014, Li *et al* 2021, Keough *et al* 2024). We note that the findings presented here are limited to motor cortex-based iBCIs and may not generalize to iBCIs using recordings from other brain areas or BCIs that use other types of electrodes, implanted or not.

One potential explanation for the lack of drop in performance is that participants ignored the *N*-Back in favor of the BCI task. However, we confirmed that the participants were engaged in the *N*-Back task, both when performing the *N*-Back by itself and in conjunction with the BCI task, by measuring accuracy, which was above 85% in all *N*-Back conditions. This indicates a high-level engagement with the *N*-Back task, and that the consistent BCI performance is not due to ignoring the cognitive task during the dual task conditions. Participants subjectively reported that the dual-tasking conditions required more mental effort (rated from 1–10) than the BCI only condition or the single tasking conditions. For P2, both BCI + 1-Back and BCI + 2-Back required more mental effort compared to 1-Back only (Fig S2). For P4, BCI + 2-Back required more effort than 1-Back only and BCI Only.

It is interesting to note that the participant whose performance did change slightly, P2, has been implanted for nearly ten years and has experienced an expected decline in signal quality (Sponheim *et al* 2021) and overall BCI performance. We quantified the average firing rate of each channel across each session for each participant. For P2, only 68 channels on average had a firing rate above 2 Hz compared to an average of 186 channels (range: 184–189) for P4’s. Additionally, we calculated the non-cross-validated decoder *R*^2^ (how well predicted kinematics match actual kinematics) for each session’s partial closed-loop calibration (see Task Design section for details). The average *X* and *Y* translation velocity *R*^2^ for P2’s sessions was 0.509 (range: 0.345–0.750), whereas P4’s was 0.856 (range: 0.665–0.920), showing the difference in movement-related information that results from the difference in signal quality. It is possible that this more degraded performance and signal are more vulnerable to the dual-tasking compared to P4. This is supported by the findings of Emami and Chau (2020), who found cognitive distractors to impact low performing participants negatively but not high performing participants. It is intuitive to assume that lower performing BCIs require more concentration to maintain performance.

### 4.2. Correlates of attention change as attentional load increases

In this study, we observed a complex relationship between EEG theta power and attentional load. In previous dual-tasking studies, theta power has been found to increase compared to single tasking (Ozdemir *et al* 2016). Although we also saw this effect, it was not consistent across conditions. P4 displayed an increase only in BCI + 2-Back, the highest load condition. This could imply that BCI + 1-Back was simply not complex enough to induce altered theta in P4 despite the subject reporting it required more mental effort than BCI Only. In the case of P2, the 1-back condition led to the highest theta band power with or without concurrent BCI control.

Alpha power exhibits a similar trend in P2 for both dual-tasking and single tasking. However, this goes against our initial hypothesis of alpha decreasing during attentional load. Alpha and theta band EEG power both follow an inverted U-shaped trend in P2. Similar trends in neural metrics of cognitive load have been seen in single-tasking contexts. One study measuring prefrontal cortex activation in fMRI during an *N*-Back, ranging from *N* =1 to *N* =6, found that activation was highest at *N* =3 but then proceeded to decline (Lamichhane *et al* 2020). One interpretation is that this reflects a shift in processing strategy where the brain regions that are active shift to handle the increasing load.

In the literature, alpha power often decreases during dual-tasking and attentional tasks (Kahya *et al* 2022). Our study observed an increase, but this is not unique. A previous study of mental workload during operation of a P300 EEG-BCI found increased alpha during dual-tasking (Käthner *et al* 2014). As in our study, their secondary cognitive tasks were presented through audio, and they noted that auditory stimulation has been found to increase alpha activity. They also discuss the alpha inhibition theory (Klimesch *et al* 2007), which states that alpha increases to inhibit non-essential brain regions to maintain task performance. İşcan & Nikulin (2018) also found that alpha power was negatively correlated with performance when BCI users had to listen to verbal counting (performance decreases with increasing alpha power), attributing the increase in power to attempts by participants to inhibit the distraction sounds. Overall, it is possible that the increased alpha in 1-Back is due to participants attempting to inhibit auditory-related stimuli so as to better handle increasing attentional load.

### 4.3. Movement-related activity remained stable under attentional load

We initially hypothesized that the changes in performance and the motor signal would occur in response to increased attentional load, with the latter providing a potential mechanism for why attentional load impacts performance. Overall, the magnitude of changes observed in movement-related activity was very small in magnitude, though some were statistically significant. P2 had a slightly lower intracortical LFP Beta band power in the BCI + 2-Back condition. P4 had a slightly higher firing rate in the BCI + 2-Back condition. The fact that the strongest motor signals were observed in the BCI + 2-back condition could indicate that some compensation occurred to maintain performance under higher load, but this was fairly minimal.

### 4.4. Limitations

One limitation of this study is that we relied on EEG to measure attention. Although this is common in the literature, the potential for variability and noise may obscure some effects of attentional load. One of the participants did indeed have a good amount of noise that necessitated removing many trials. Another limitation is that the BCI task could have been too simple to show large performance effects with dual-tasking even though the BCI task was representative of typical BCI use and more complex than a standard center-out paradigm. Future studies should ensure the tasks are sufficiently challenging to fully sample the range of possible attentional load conditions. Finally, this study is limited to 2 participants due to the nature of iBCI trials that have inherently small sizes given the need for surgery (Hochberg *et al* 2012, Ajiboye *et al* 2017, Willett *et al* 2021, Card *et al* 2024). However, both participants support the conclusion that iBCI is robust to dual-tasking despite the performance and signal quality differences that derive from each having different implant lengths. Both also exhibited an increased attentional load during dual-tasking but maintained stable movement-related neural activity across dual-tasking conditions. While the participants differed in the extent to which each dual-tasking condition impacted their attentional load, it is encouraging that both demonstrated consistent performance. Larger studies may be needed to fully characterize the range of responses across individuals and tasks.

## 5. Conclusion

This work adds to the small literature investigating the impact of attentional load on BCI performance. It is one of very few studies that have looked at the impact of attention or distraction during iBCI control (Stavisky *et al* 2020, Guthrie *et al* 2021). Unlike these previous studies, neural correlates of attention were quantified with whole brain EEG measures. The *N*-Back was sufficient to drive changes in attentional load, but dual-tasking with BCI resulted in very minimal changes in performance. iBCI cursor control was robust to changes in attentional load, but more complex applications may be more susceptible to attention-related neural changes.

## Data Availability

The data that support the findings of this study are available in the Data Archive for the BRAIN Initiative at the following URL/DOI: https://doi.org/10.18120/ar71-yw82 (Canario *et al* 2026). Supplementary Figures available at https://doi.org/10.1088/1741-2552/aebb65/data1.
